# Maternal request caesareans and COVID-19: the virus does not diminish the importance of choice in childbirth

**DOI:** 10.1136/medethics-2020-106526

**Published:** 2020-09-10

**Authors:** Elizabeth Chloe Romanis, Anna Nelson

**Affiliations:** 1 Centre for Ethics and Law in the Life Sciences, Durham Law School, Durham University, Durham, UK; 2 Centre for Social Ethics and Policy, Department of Law, The University of Manchester, Manchester, UK

**Keywords:** autonomy, feminism, reproductive medicine, right to healthcare

## Abstract

It has recently been reported that some hospitals in the UK have placed a blanket restriction on the provision of maternal request caesarean sections (MRCS) as a result of the COVID-19 pandemic. Pregnancy and birthing services are obviously facing challenges during the current emergency, but we argue that a blanket ban on MRCS is both inappropriate and disproportionate. In this paper, we highlight the importance of MRCS for pregnant people’s health and autonomy in childbirth and argue that this remains crucial during the current emergency. We consider some potential arguments—based on pregnant people’s health and resource allocation—that might be considered justification for the limitation of such services. We demonstrate, however, that these arguments are not as persuasive as they might appear because there is limited evidence to indicate either that provision of MRCS is always dangerous for pregnant people in the circumstances or would be a substantial burden on a hospital’s ability to respond to the pandemic. Furthermore, we argue that even if MRCS was not a service that hospitals are equipped to offer to all pregnant persons who seek it, the current circumstances cannot justify a blanket ban on an important service and due attention must be paid to individual circumstances.

## Introduction

COVID-19 has had a considerable impact on pregnancy and birthing services. The latest concern is that some hospitals have introduced blanket policies to deny all maternal request caesarean sections (MRCS) during the pandemic; reports indicate that Milton Keynes University Hospital has instituted new guidance to refuse all MRCS.^1^ MRCS is the provision of a caesarean section on the request of a pregnant person^i^ for any reason—sometimes in the absence of obstetrical indications. National Institute for Heath and Care Excellence (NICE) guidance^2^ and the law^3 4^ are supportive of pregnant people’s choice about/in childbirth, including MRCS. In this paper, we argue that even during a pandemic the *routine* denial of MRCS is inappropriate. First, we outline that MRCS is a health matter and that pregnant people have the right to make choices about childbirth. It is unfortunate that MRCS has consistently been characterised as an ‘elective’ procedure, and this has been reinforced by the refusal of some trusts and hospitals to provide this care during COVID-19, when in reality for many pregnant people it is necessary to preserve their physical or mental health. Second, we highlight how the pandemic has potentially limited access to MRCS and why this is concerning. This has been a significant real-life problem for a number of people giving birth during the pandemic, and unless addressed there is reason to believe it might again be an issue in future. Third, we demonstrate that arguments about health or resource allocation cannot be used to deny wholesale the provision of MRCS.

While changes in pregnancy and birthing services during the pandemic have resulted in limitations on choice in childbirth in a multitude of ways,^5 6^ we limit our scope to the impact of the crisis on the provision of MRCS in response to the recent news.^1^ MRCS often captures significant public attention as it is considered a ‘controversial’ practice^7^ both by the public and by some medical professionals.

## MRCS: autonomy and health

MRCS is often mischaracterised as ‘irrational’^8^ or the result of misogynist narratives about the vagina and male sexual gratification.^9^ However, most pregnant people seeking MRCS do so for health reasons^5^; often do due to an underlying health condition that, while not meeting the threshold of severity to make caesarean a clinical *necessity*, is still significant and might be impacted by vaginal delivery (VD), for example, symphysis pubis dysfunction^10^ or digestive disorders.^4^ MRCS also encompasses instances in which an emergency caesarean could become necessary if a planned caesarean is not undertaken earlier.^10^ Furthermore, what constitutes ‘clinical need’ for caesarean is a matter of clinical discretion—some doctors believe that the provision of caesarean is appropriate to provide assistance to any persons who are concerned about the risk of pelvic floor disorders after VD.^11^ Rebecca Schiller at Birthrights explains that the people the charity supports have endured ‘previously traumatic births, physical or mental ill-health or survivors of sexual abuse. Others have carefully examined the evidence available… and made personalised decisions that a planned C-section will give them and their baby the best chance of an emotionally and physically healthy start’.^12^ It is inappropriate to describe MRCS as ‘clinically unjustified’.^7 13^

The potential impact of birth on the body of the person who gives birth is substantial. Both caesareans and VD take time to recover from, the former is a surgery and the latter is physically exhausting. Further, both can result in scarring. It is a fundamental principle of English law that a person is entitled to determine what happens to their own body, and consent to or refuse any medical intervention or treatment.^14^ Pregnant people thus should be entitled to determine their mode of childbirth as it is a physical process that impacts on their body immediately and in the long term. We believe that ‘denying choice in childbirth means denying… [pregnant people] the opportunity to experience some empowerment and take ownership of pregnancy, thus mitigating some of the difficulties unilaterally imposed on bodies with female physiology in sexual reproduction’.^7^

Second, health indications for caesarean can be much broader than an immediate physical health need, and in considering clinical indications in childbirth health should be viewed holistically.^4 7^ The most common reason MRCS is sought is because of previous birth trauma.^10^ Other common motivators include underlying health conditions (both related and unrelated to pregnancy), previous sexual assault, primary tokophobia (fear of childbirth) or general anxiety around childbirth.^10^ These all represent instances in which not allowing a pregnant person to opt for MRCS could have a significant impact on their mental health while awaiting their childbirth, during and for a long period after (indeed, any trauma experienced in the process may have a permanent impact on sense of self). It is important that the mental health of people giving/who have given birth is afforded due respect. It is inappropriate to *solely* characterise MRCS as ‘elective caesarean’ because many pregnant people seek this for reasons related to their health, and because in many instances MRCS is undertaken to avoid the risk of emergency caesarean becoming necessary during a planned VD.

Denial of choice in childbirth can cause significant trauma and have long-term implications for postbirth mental health. Considerable anxiety will be experienced by pregnant people unsure whether their desire for MRCS will be facilitated, and it is notable that stress during pregnancy is associated with preterm birth.^15^ ^ii^ Childbirth is a hugely significant event in the life of most who give birth; the potential (long term) impacts for those whose delivery is marred by loss of control, lack of dignity and denial of choice must not be underestimated. Removing the autonomy to define what this potentially life-altering experience will look like for themselves ‘forces… [pregnant people] to relinquish their identity’^4^ and can be profoundly distressing. The Birth Trauma Association—a charity supporting people who suffer postbirth Post-traumatic stress disorder (PTSD)—identifies ‘feelings of loss of control’ and ‘lack of dignity’ and ‘not being listened to’ as factors which make this PTSD more likely.^16^ A negative birth experience can also severely erode confidence in the health system (and specifically pregnancy and birthing services); affecting not only that person during any future pregnancies but also contributing to a climate of doubt, as the person shares their negative experiences with others.^17^

### Risk and MRCS

The most common objection raised by critics of MRCS is that it is riskier than VD and since these risks are unnecessary, they should be avoided.^18^ However, there is not clear evidence to indicate that MRCS is *significantly* riskier than VD. The evidence relied on to make this objection uses data which conflates outcomes of MRCS and *emergency* caesarean (which is more common).^19^ There are therefore significant confounding variables in existing data, including the potential underlying emergency circumstances that indicated caesarean.^20 21^ We argue that MRCS is likely to be much safer than these data suggest because of the absence of emergency conditions,^20^ the implementation of effective safety protocols,^21^ and reduction in circumstantial stress felt by attending medical professionals and/or distress in the pregnant person.^4^

This argument about risk is also entirely devoid of examination of an individual person’s circumstances and health and this is inappropriate. No childbirth is entirely ‘risk free’.^4^ While some risks are more commonly associated with MRCS (eg, those related to anaesthetics and a longer hospital stay^20^), other risks are more commonly associated with VD (eg, pelvic floor disorders^11^ and complications resulting from the use of forceps or vacuum^22^). The gravity and significance of different risks to individuals will vary. As such, pregnant people ‘should be entitled to decide which physical impacts they might experience [in childbirth] and which risks to assume’.^7^ Even if there were evidence that MRCS was *significantly* riskier than VD for a particular pregnant person it is for that person, appropriately counselled, to decide which risks they would rather assume.^4 7^

### MRCS policy and law

NICE guidance indicates that ‘if after discussion and offer of support (including perinatal mental health support for… [pregnant people] with anxiety about childbirth), a vaginal childbirth is still not an acceptable option, offer a planned CS’.^2^ The guidance is supportive of choice in childbirth, recommending that MRCS be provided after informed counselling of the overall risks and benefits of MRCS compared with VD.iii It has also been argued *Montgomery* 3 necessitates that MRCS should be discussed with patients—as part of the obligation to disclose and discuss ‘reasonable alternatives’ to a proposed treatment.^4^

While the law remains clear that a doctor cannot be compelled to perform MRCS absent emergency circumstances,^23^ the NICE guidance instructs doctors that if they are uncomfortable providing MRCS to a person who finds VD unacceptable, they must refer the patient to a colleague.^2 4^ Thus, while the law and NICE guidelines do not afford a pregnant person the right to demand that a doctor perform MRCS, they do afford the right to be appropriately counselled through reasonable alternatives (including MRCS) in childbirth. Despite this clear position, there remain several trusts that do not offer MRCS,^10^ such that access to the procedure remains a lottery in the UK.^4^ We believe this to be a matter for concern as it contributes to the characterisation of MRCS as wholly elective and it has been exacerbated by decisions that some trusts and hospitals have made during the pandemic, as we will discuss in this paper.

The Royal College of Obstetricians and Gynaecologists (RCOG) advises that there is no evidence amid COVID-19 to recommend or prefer one mode of birth over another—the only exception being if a pregnant person is in respiratory distress because of COVID-19 infection. RCOG COVID-19 guidance stipulates that ‘mode of birth should be discussed with the… [pregnant person], taking into consideration her preferences and any obstetric indications’.^24^

## MRCS during a pandemic

The importance of choice and individualised, holistic conceptions of health in childbirth is not diminished by the COVID-19 crisis. The current crisis has exemplified the need to embed choice into pregnancy and birthing services. Birth can be empowering and beautiful where the pregnant person is centred and supported in making choices, but equally traumatic and harmful to a person’s body and well-being if birth involves feelings of compulsion or being subjected to the control of others.^7^ Day-to-day life is more difficult for most due to the current restrictions on daily activities. It seems, prima facie, wrong to deny *all* pregnant people MRCS in a situation in which a traumatic birth may be *even more* difficult to recover from.

Pregnant people have raised concerns about whether their birth partner will be allowed to be present while they are giving birth in a hospital^5 6 25^; concerns rooted both in reports around certain hospitals' restrictive policies on the matter, and in the individual circumstances of some pregnant persons which means that their birthing partner could not be present (eg, other caring responsibilities).^26^ For some pregnant people concerned about VD because of, for example, primary tokophobia or a previous traumatic experience, giving birth alone or without their usual support networks could exacerbate their concern, anxiety, or the harm that they may experience. Even where pregnant people have support *during* a difficult birth, the reduction in social resources (such as visits from friends and family)^iv^ following this experience may make recovery more challenging.^v^ Furthermore, the lack of face-to-face birth counselling, important for many people experiencing tokophobia who want to have a VD,^2^ may mean some pregnant people feel that they are unable to ready themselves. It is understandable then that some pregnant people may feel that MRCS is preferable for them in the current climate. These wishes should be respected where possible for the same reasons that we should value choice in childbirth when pregnancy and birthing services are business as usual—to respect pregnant people’s bodily autonomy, to afford them dignity and to ensure that their health and well-being is secured during and after birth.

Because of the importance of choice in childbirth,^7^ we believe that the possibility that MRCS is *blanket* denied by some hospitals without consideration of individual circumstances is concerning. Such a policy would require a strong justification. The reasons that might be advanced to claim that MRCS becomes less safe or impossible during a pandemic are unconvincing. Moreover, we will show that even if they were persuasive, potential objections cannot be demonstrated to be universally true meaning that they cannot provide justification for ceasing *all* MRCS.

## Increased exposure to infection?

Since MRCS does usually necessitate a longer hospital stay^20^ than VD, it might be argued that it is inadvisable during a pandemic. It may increase a pregnant person’s exposure to COVID-19 infection because of a longer stay in a higher risk environment.

Similarly, the need for collective effort to effectively tackle the pandemic might lead some to argue against MRCS based not on individual risk, but on a more utilitarian public health basis. The suggestion here is that allowing an individual to make a decision which may expose them to unnecessary risk of infection, such as opting for MRCS, is problematic because it leads to increased risk of that person becoming a ‘transmitter’ once they leave hospital.

We believe that if appropriately managed and if PPE requirements are in place the number of pregnant people that catch coronavirus in hospital can be minimised. Moreover, exposure is realistically unlikely to be increased significantly by extra days in hospital—since it will be the protocols that are in place in the hospital that have the most measurable impact. We note that at as many homebirthing services were suspended in the UK during the pandemic,^5 6^ there was an increased expectation that people attend hospital during birth; we can only assume that this is indicative of a belief by medical professionals that hospitals did not pose an unreasonable risk of infection. Though there is, plainly, a longer window for exposure in instances where a person remains in the hospital for longer, we do not perceive this to be a significant problem as the risk of exposure in the maternity wards (given all the additional measures which have been implemented) is not unreasonable.

Importantly, we stress that decisions about their health are for pregnant people themselves to make. Pregnant people should be advised about the potential risks of COVID-19 exposure in hospital as part of the process of properly informed counselling about choices in childbirth,^7 27^ but ultimately it is for them to weigh this new potential risk attributable to MRCS against the risk of VD. This may change the perspective of some pregnant people; however, there are people requesting MRCS for (physical or mental) health reasons who may still believe that MRCS, on the balance of all the risks and evidence^7^—remains the right choice for them. Any potential blanket restriction on MRCS because of the pandemic (even at a local level) is problematic because it reinforces the existing lottery in the extent to which pregnant people are empowered to be active participants in choice about their childbirth,^4^ and prevents individuals from making the right choice for them in their circumstances.

The point might be raised here that it is not only risks to pregnant people in terms of increased exposure to infection that we should be concerned about; we ought to consider the potential risks to staff from MRCS during a pandemic. We note, however, that those staff that are involved in MRCS may also be involved in the care of pregnant people who are delivering (vaginally) in hospital and so one would imagine there might be little difference, at least for some staff, in their exposure. It could be that risk profiles are different in the operating theatre compared with a labour room because of the need for more staff,^vi^ or their need to work in closer proximity, so there may be grounds to limit MRCS. We do not believe, however, that such considerations can justify a blanket ban. Such an approach removes the necessary nuance from decisions about mode of delivery and does not provide scope to recognise that there will be instances where the needs of a certain pregnant person should outweigh a potential increase in the risk profile of staff. For example, where it seems that an emergency caesarean might be likely (because of underlying health conditions) if MRCS is refused, or because the likelihood of severe birth trauma poses a major risk to the pregnant person’s well-being. On these accounts we note that, even during a pandemic, there is a responsibility on health professionals to provide the appropriate care where it is necessary to avoid serious injury to a person’s health. Precautions can also be carefully thought through and taken when MRCS is scheduled. In those instances where emergency caesarean is potentially likely if MRCS is refused, the risk profile of the procedure for staff may increase because of the need for general anaesthetic (as it is aerosol-generating),vii and because emergency conditions might make safety precautions more difficult to plan and undertake.

## Resource allocation

One of the most common objections raised to MRCS is that it should not be publicly funded, as the procedure requires additional resources and associated costs compared with VD.^22 28 29^ In the context of the pandemic, it might be argued that any additional costs for elective procedures are inappropriate while the National Health Service is responding to an emergency, or that the provision of an MRCS might divert resources away from the ‘front line’ of the fight against coronavirus. We believe these claims are false; financially, MRCS is not actually particularly more expensive, *especially* when the mental health implications and related costs are taken into consideration. However, even if MRCS was slightly more costly in financial terms, the implications of denying pregnant people’s choice in childbirth are far *more* costly in a broader (and more important) sense. It is unfortunate that resource issues are so centred in debate about MRCS—the impact on the pregnant person must be considered central and is more important than any difference in care costs. We do, however, here address the concerns about cost that we believe are misplaced.

### The comparative cost of MRCS

Prima facie, caesareans do appear to cost significantly more than VD; however, this cost-analysis loses its persuasive force once the *method* of birth stops being considered in silo and is instead considered in a holistic context of the birthing experience.

Well-managed and uncomplicated MRCS should not be assumed to be comparable in cost to an emergency caesarean. The cost of an emergency caesarean is significantly higher, at approximately £3820 compared with £2922 for MRCS.^30^ Furthermore, the cost forecasting for planned VD should also take the cost of potential emergency caesarean into account. This is important because MRCS is often sought by pregnant people because of underlying health conditions that increase the likelihood that emergency caesarean may become necessary if MRCS is not scheduled. The occurrence of emergency caesarean in those instances in which MRCS is refused also challenges the validity of a resource-based opposition to MRCS. Birthrights also note that once the cost of treating urinary incontinence which results from VD is considered, the difference in the cost of these birth methods drops so significantly (from about £700 to only £84) that NICE have indicated is insufficient to influence the decision making process.^10^ Significant resources are deployed in managed VD (including analgesics, medical supervision and equipment)^4^ and urinary incontinence is only one of the potential side effects of VD that might require subsequent treatment. Thus, the difference in cost is likely to be even more negligible. MRCS could be similar in cost to, or less costly than, technologically assisted VD.^31^

Even if it were numerically accurate to state that MRCS was significantly more costly than VD as a *birth method—*this fails to acknowledge the wider consequences, and therefore does not represent an accurate cost analysis. Where resource-based arguments are advanced as reasons to reject MRCS, insufficient recognition is given to the mental health implications of refusing MRCS and the contingent costs, as well as the potential costs of liability in negligence that might arise in some instances. The National Health Service (NHS) litigation authority identifies that the highest value negligence claims are against obstetricians,^32^ and their report published in 2012 emphasised the importance that should be placed on the NICE guidelines and pregnant people’s choice in delivery.^32^ viii Over 25% of negligence claims in the context of childbirth concern inadequate information about choice in delivery,^32^ including recent high-profile litigation.^3^ While these conclusions result from a report that is, at the time of writing, 8 years old, engagement with organisations like Birthrights and the volume of litigation on choice in childbirth continues to evidence that this problem remains prevalent today. West *et al* concluded in 2018 that MRCS could be around £439 per birth cheaper than planned VD when the value of claims for obstetric harm are factored in.^ix^ Therefore, they conclude, there is no justification to deny MRCS on the grounds of comparative cost.^33^ It is evident that a failure to counsel about MRCS or the refusal of genuine requests may be substantially more costly than performing these surgeries.

A failure to appropriately attend to choice in childbirth can cause significant birth trauma and mental health conditions that require subsequent and continuing treatment.^16^ These difficult conditions may be *even more* impactful on a person’s life at this time as the pandemic has left individuals feeling increasingly isolated. The pandemic is likely to cause a significant increase^34^ on the burden faced by already incredibly stretched mental health services. The Centre for Mental Health have analysed data on mental health following previous epidemics and the 2008 financial crisis and indicated that a substantial increase in the number of people experiencing mental health difficulties over the next year is likely.^35^

### MRCS during COVID-19

It is often pointed out that increasing numbers of caesareans—to which MRCS contributes^x^—increases bed occupancy in hospital. The potential for MRCS to lead to ‘postnatal bed blocking’^30^ is advanced by some as a reason why MRCS is undesirable, or even unachievable. However, research indicates that the increasing demand for postnatal beds is not significantly attributable to the increase in caesareans—largely due to ‘the marked decline in the average length of stay post-CS’.^30^ It might be argued that, during the pandemic, any intervention increasing the likelihood of a hospital stay should not be facilitated as it increases the likelihood that intensive care beds may be occupied by people following MRCS complications, when these beds are necessary for those suffering from COVID-19. We believe it highly unlikely that the provision of MRCS will significantly divert resources from the ‘front line.’

We acknowledge that ‘bed blocking’ is not the only resource-based issue associated with MRCS. This is a surgical procedure which therefore requires access to an operating theatre, sterile equipment, scrub nurses and an anaesthetist.^xi^ However, MRCS is not a hugely popular choice among pregnant peoplexii—data from NHS England reported that for every month between September 2018 and January 2020 MRCS accounted for 16% of deliveries across the country.^36^ xiii It seems unlikely there would be a big increase in demand during the pandemic. If MRCS were performed at this rate, it would not account for a substantial drain on staff time in individual hospitals (assuming that these deliveries are not concentrated).xiv Furthermore, the incidence of complications severe enough to require admission to intensive care after MRCS is low.^21^ It is therefore unlikely that MRCS provision could be happening on such a scale as to take significant number of anaesthetists and other staff away from dealing with patients with COVID-19. In any event, such an argument cannot be justification for a blanket ban on the procedure. Even if the rate of MRCS was to increase and/or it was impossible to facilitate every MRCS requested, there will be cases where MRCS is necessary to prevent grave and permanent damage to the pregnant person’s physical and mental health and it could (and should) still be provided in these instances without a substantial impact on a hospital’s resources.

MRCS is a *time-critical* surgery with profound benefits for some pregnant people’s health and well-being. It might be considered comparable to some other emergency surgeries (that continue during the pandemic for obvious reasons). Furthermore, some of the more time sensitive services (including some surgeries),^37^ that were initially suspended have—at the time of writing and of revising this manuscript—begun to resume in some parts of the UK.^38^ This is as the number of COVID-19 cases requiring hospitalisation has continued to fall, and thus resources are available to resume time-sensitive services. There can, therefore, be no reason to suppose that there is not the capacity for MRCS. A blanket restriction on MRCS cannot be justified by resource allocation arguments—even during COVID-19.

## MRCS, human rights and equality

The strategy of our argumentation thus far has been to respond to issues that might be raised to justify a restriction on choice in childbirth in the specific circumstances presented by the COVID-19 pandemic. However, we believe it is important to end this paper by reaffirming that regardless of pandemic conditions there is no justification for blanket restrictions on pregnant people’s access to MRCS as part of their right to choice in childbirth and to preserve health.^4 7^ Access to MRCS is incredibly important for some pregnant people. Disregarding choices in childbirth can cause extensive and long-lasting harm.

Though a full exploration is beyond the scope of this paper for reasons of space, it is important to recognise that there are powerful human rights and equality arguments that reinforce the necessity of providing MRCS in general and for continuing to do so during COVID-19.^xv^ While we recognise that health services have extremely difficult decisions to make during COVID-19, it is both ethically and legally necessary to ensure that the rights of people giving birth remain protected at this time—and blanket bans are an inappropriately blunt tool for this very nuanced task. Blanket bans on health services prevent individualised decision making based on patient needs and are potentially incompatible with the State’s obligations under the European Convention on Human Rights. We do not have space in this paper to consider the legalities of this but raise this matter for further reflection. The right to private and family life has been found to be inclusive of choices about birthing.^39^ Moreover, given that a number of the people seeking MRCS may be doing so because of longstanding health conditions, ensuring their access to appropriate medical care in pregnancy and birthing should be thought of as an important part of ensuring that they are not discriminated against in the care that they receive. This remains important during a pandemic.

While a pandemic undoubtedly introduces the need to make difficult public health decisions, these decisions must be carefully balanced and must continue to protect the health and human rights of pregnant people.

## Conclusion

Choice in childbirth is crucial for pregnant people’s physical and mental health and remains important during COVID-19. NICE guidelines and the law are both broadly supportive of MRCS counselling and of pregnant people being able to opt for MRCS. MRCS is not clinically unjustified and can be crucial to ensure a pregnant person’s individual health, and that their autonomy and dignity are respected. Characterising MRCS as wholly an ‘elective procedure’ is often inaccurate because of the reasons that MRCS is often sought by pregnant persons preparing to birth. We argue that MRCS remains an important choice that should be available during the pandemic. While there may be some instances in which it is not possible to facilitate MRCS, this is unlikely to necessitate any *blanket* policy that MRCS should not be performed. Furthermore, we considered some of the objections that might be raised to MRCS—namely related to pregnant people’s health and to resource allocation. These objections are insufficient to justify restrictions on MRCS. It was important to address this issue because restrictions on MRCS continue to impact on pregnant people’s health, and this has been exacerbated during the pandemic. As we see restrictions easing and hospitals increasing the health services, they are providing it is important that MRCS is facilitated, and it is important that we reflect on the damage caused by restricting these services ahead of any future peak in COVID-19 cases or a future pandemic. It is the case that restricting MRCS will have been harmful to individual pregnant people; moreover, it has contributed to the continued perpetuation of MRCS as ‘wholly elective’ which is not appropriate or accurate.

When a holistic approach is taken to well-being in and after childbirth, which considers the broader reality in which MRCS requests are situated, the arguments against MRCS based on resource/cost expenditure or the absence of clinical ‘indications’ are unconvincing. Where the dignity, autonomy and health of pregnant people are at stake, excessive deference to financial/resource concerns or pathologies^4 7^ is inappropriate and fails to adequately centre the rights and dignity of pregnant people before, during and after birth.

## Footnotes

We use the term pregnant person here to recognise that it is persons with female physiology who carry pregnancies. We note that while the majority of pregnant people identify as women, this is not always the case and we believe that it is important to use language that is inclusive of pregnant people and those persons who birth but do not identify as women. Ross and Solinger note that using ‘women’ to describe those who are and can get pregnant is inaccurate in being too narrow (exclusionary to gender fluid, non-binary or other pregnancies) and too broad because many people who identify as women do not have the physiology to get pregnant.^40^ We do not seek to erase the fact that the vast majority of pregnant people identify as women and that the routine historical and socialised association between female biology and the societal expectations imposed on those who are, or who are perceived as ‘women,’ is one of the ways in which structural discrimination against women and female people is perpetuated. However, we believe that preventing the routine erasure of non-women who birth (who are often marginalised significantly in many aspects of healthcare) to be important.

Preterm birth is also associated with structural disadvantage including socioeconomic circumstances and ethnicity. It is important to note that there has been an observably higher impact of COVID-19 on black people, Asian, and minority ethnic persons during the pandemic, and this may exacerbate existing inequalities experienced by black, Asian and minority ethnic people in accessing pregnancy and birthing services in the UK.^41^

It is unfortunate that some of the language in this paragraph of the guidance does sound as if the doctor’s role in counselling is to try and dissuade a pregnant person from MRCS—and to only permit MRCS only in instances where vaginal birth is ‘unacceptable’ to the pregnant person, rather than a preference. However, the guidance remains supportive overall of the choice to opt for MRCS.

This remains true whether the person recovers at home or in the hospital. Most NHS Trusts have put in place policies which prohibit or severely limit visitors to labour wards, and at present the COVID-19 guidance and legislation still prohibits people from visiting others in their home.

One of the anonymous reviewers observed that extra time in hospital at this time due to a surgical procedure might potentially exacerbate feelings of isolation among some people who are giving birth. This is a valid point and we believe that this is something that will need addressing—both in terms of being raised with pregnant people who are making decisions about MRCS and in terms of ensuring that there is adequate postpartum mental health support available.

We are grateful to an anonymous reviewer for raising this point.

We are grateful to an anonymous reviewer for raising this point.

These conclusions are obviously focused on action that professionals should take in order to reduce the number of incidents that give rise to negligence claims—we are grateful to an anonymous reviewer for encouraging us to highlight this.

It must be noted that this study has not been peer reviewed.

It has been argued that MRCS is not the cause of, or a significant factor in, rising caesarean rates.^7^

We are grateful to an anonymous reviewer for highlighting this point.

We note that even if this is not a popular choice made by people giving birth, this does not diminish the force of our arguments about the importance of this choice being available for those pregnant persons who believe that MRCS is the right course of action for them in their particular circumstances.

November 2019 was an outlier in this period, as the estimated percentage of elective caesarean performed at this time dipped to 13%.

There is consistently a slightly higher percentage of elective caesareans taking place in the South East compared with other regions of England.

We explore these themes in coauthored work regarding homebirth that is forthcoming elsewhere.^6^
